# A functional movement screen profile of an Australian state police force: a retrospective cohort study

**DOI:** 10.1186/s12891-016-1146-0

**Published:** 2016-07-18

**Authors:** Robin Marc Orr, Rodney Pope, Michael Stierli, Ben Hinton

**Affiliations:** Tactical Research Unit, Bond University, Gold Coast, QLD 4226 Australia

**Keywords:** Law enforcement, Tactical, Screening, Movement skills, Injury

## Abstract

**Background:**

Police officers are required to perform dynamic movements in unpredictable environments, the results of which can lead to injury. Early identification of poor movement patterns of a police population, and potential sub groups within this population, may provide opportunities to treat and minimise injury risks. The aim of this study was to profile the functional movement capabilities of an Australian state police force and potential sub groups through a retrospective cohort study.

**Methods:**

Retrospective data from an Australian State Police Force were provided for analysis (♂ *n* = 1155, mean (±SD) age = 31.34 ± 8.41 years: ♀ *n* = 357, mean age = 27.99 ± 8.02 years). Data consisted of Functional Movement Screen (FMS) assessment results of male and female trainees and qualified police officers with all assessments conducted by a qualified Police Physical Training Instructor.

**Results:**

Significantly higher (*U* = 253863, *p* < .001) FMS total scores were found for recruits (mean 15.23 ± SD 2.01 points) when compared to attested officers (14.57 ± 2.96 points) and differences in FMS total scores also approached significance for females (15.24 ± 2.35 points) when compared to males (14.84 ± 2.55 points, *U* = 186926, *p* = .007), with age found to be a key, significant factor in explaining these observed differences (F (1,1507) = 23.519, *p* < .001). The FMS components demonstrating poorest movement performance across all groups were the hurdle step and rotary stability.

**Conclusions:**

Generally, police personnel (both attested officers and recruits of both genders) of greater age have a lower functional movement capability when compared to younger personnel, with greater percentages scoring 14 or below on the FMS. Specific conditioning programs to improve strength, range of motion and stability during identified key movement types in those demonstrating poorer movement performance may serve to reduce injuries in police personnel.

## Background

Police officers are required to perform tasks that can include dynamic movements like running, jumping, crawling, balancing, climbing, lifting, carrying, pushing, pulling, fighting and dragging, in unpredictable environments [1, 2]. The results of these actions can lead to injury of the back, knee and shoulder - known bodily sites of injury in police officers [3]. Early identification of poor movement patterns that are associated with performing these tasks may provide opportunities to treat and minimise injury risks for police officers.

One means of identifying poor movement patterns is through the use of the Functional Movement Screen (FMS) tool, a tool with a high inter-rater reliability (ICC_2,1_ = 0.74, 95 %, CI = 0.60, 0.83) [4] and intra-rater reliability (ICC_2,3_ = 0.75, 95 % CI = 0.526 to 0.872) [5]. The FMS is an evaluation tool that consists of seven movement patterns used to assess an individual’s movement in a dynamic and functional way [6, 7]. Although limited, research does suggest the potential for poor execution of the specific FMS elements to be associated with an increased risk of musculoskeletal injury [6, 8]. As such, the FMS tool offers an approach to injury prevention and movement performance prediction by identifying an individual’s functional limitations and/or asymmetries [5–7, 9, 10]. These limitations/asymmetries may then potentially be addressed by targeted physical training interventions.

The use of total FMS scores as a predictor of injury forms one of the key tenets for its use within physically active populations [6]. Previous studies have suggested that low total FMS scores, specifically those less than or equal to 14 (out of a possible 21), have an association with musculoskeletal injuries in athletic [7, 11], general [10, 12], and tactical [13, 14] populations. In one study with National Football League players it was concluded that players with total FMS scores ≤14 had an 11-fold increase in risk of injury when compared to players with scores >14 [7]. These findings of an increased risk of injury with total scores ≤14 are supported by research in other athletic [11] and tactical populations [13, 14] as well as the general population [10, 12].

While evidence is available for the use of the FMS as a predictor of injury, there is currently limited evidence that the FMS can predict occupational performance – that is the ability to perform daily work tasks [15]. Considering this, the FMS assesses fundamental movement patterns of an individual in a dynamic and functional way - movement patterns typically performed by police officers as part of their occupation [1, 2]. As such, the question arises of whether this tool could be employed to assess occupational capability in tactical personnel.

As a first step in exploring the potential application of the FMS tool in police populations, the objective of this study was to profile functional movement capabilities of police officers, and so to determine their risk profiles based on the findings of prior FMS research. Differences in FMS scores between new recruits and attested (fully qualified) officers and between female and male populations were examined, as previous research has suggested that trainees are more likely to be injured than qualified tactical personnel [16] and female personnel are more likely to be injured than male personnel [17, 18]. Population-level profiles of these types are required to better inform future training and injury risk management initiatives, return-to-work rehabilitation guidelines, and research in the police context.

## Methods

The study employed a cross-sectional study design, in which FMS scores were assessed once only in consenting recruits and attested police officers, with data collected over a 30 month period (January 2012 to July 2014). The setting for this research was facilities of the state police force. Both recruits and attested officers were selected in this study to ensure a complete representation of the police force from initial training to full time service.

The participants included recruits who underwent FMS testing as a routine part of the induction protocol into the health and fitness component of their recruit training. All of their data used in this study was captured initially for physical training purposes but was subsequently provided to the researchers in a non-identifiable form for use in the study. The participants also included attested officers who were offered the FMS on a volunteer basis in the Local Area Commands, on training days, again originally to support the development of physical training plans for these officers, but with scores provided to the researchers subsequently in non-identifiable form. A total of 1512 personnel comprised the participants for this study (see Table 1). Inclusion criteria for data records were: a) the participant completed all aspects of the FMS; and b) the police recruit participants had not attempted the police training previously. The exclusion criterion for this study was a record where a recruit or officer did not complete the FMS in its entirety, due to an injury they were suffering at that time.

**Table 1:** Demographics by cohort, status (recruit or attested officers) and gender

	Cohort (*n* = 1512)		Recruits (*n* = 823)		Attested Officers (*n* = 689)	
	Male	Female	Male	Female	Male	Female
n (%)	1155 (76%)	357 (24%)	573 (70%)	250 (30%)	582 (84%)	107 (16%)
Age (mean ± SD) yrs	31.34 ± 8.41	27.99 ± 8.02	25.50 ± 5.80	25.78 ± 5.57	34.84 ± 8.00	36.87 ± 6.88

The FMS was selected as the main outcome measure for this program of research due to its previous use as a reliable predictor of injury risk in athlete [7, 11] and tactical populations [19, 20] and its use as a return-to-work outcome measure in police rehabilitation [21]. The FMS assesses seven movement patterns that include an overhead squat, hurdle step, in line lunge, shoulder mobility, active straight leg raise, push-up, and rotary stability [6].

Each component of the FMS is scored on a scale of zero to three points. A score of zero is assigned if the participant experiences pain with any portion of the movement pattern. A score of one identifies that the participant did not experience pain but could not complete the movement pattern as instructed, while a score of two identifies that the participant could complete the movement pattern pain-free but required some level of compensatory movement pattern. A score of three identifies that the participant’s movement pattern was completed as instructed, with no movement compensation noted, and with the movement being pain-free [6]. The total FMS score is calculated by summing the scores of individual elements of the FMS and can range from zero to a total score of 21 [6].

All FMS assessments were conducted by qualified NSW Police Physical Training Instructors familiar with the FMS. For the police recruits, a single Physical Training Instructor was assigned to assess each station and the recruits transitioned through from one station to the next. For the attested officers, a single Physical Training Instructor conducted the assessment in its entirety. As the FMS has high inter-rater reliability [4] and intra-rater reliability [5], these differences in FMS assessors between recruits and officers are unlikely to have significantly influenced the study results. Furthermore, the reliability of the FMS within the tactical population has been demonstrated in previous research [4] and to further enhance reliability and standardise the testing procedure, a qualified NSW Police Physical Training Instructor (PTI), formally trained in the FMS tool at their annual PTI training and with notable experience in using the tool previously, assessed each police member. Ethics approval for this research was provided by the Bond University Human Research Ethics Committee (RO1858 and RO1670).

Demographic data and the FMS scores for each group were initially analysed descriptively, to derive frequencies and mean ranks and means, as appropriate depending on variable type. Distributions of FMS total scores in each group were plotted and then visually and descriptively compared as a basis for considering and comparing the injury and movement performance risk profiles of police recruits or officers in each group. Inferential analysis first involved Mann–Whitney U Tests to investigate differences between gender groups and recruit versus officer groups in the mean ranks of FMS total scores (as 1 dependent variable) and in the distributions of individual component scores of the FMS (comprising 7 further dependent variables). Given the likelihood of age differences between recruit and attested officer groups, differences in mean FMS total scores between groups (male vs female personnel and recruits vs attested officers) were further analysed using an Analyses of Covariance (ANCOVA), with age entered as a covariate.

The overall level of significance was set *a priori* at 0.001 for all individual tests of statistical significance following Bonferroni correction to control the family-wise error rate that would otherwise be associated with the conduct of the large number of statistical tests of significance performed within the study. Data were analysed using Statistical Package for the Social Sciences (SPSS) version 22 [22].

## Results

**Fig. 1:**
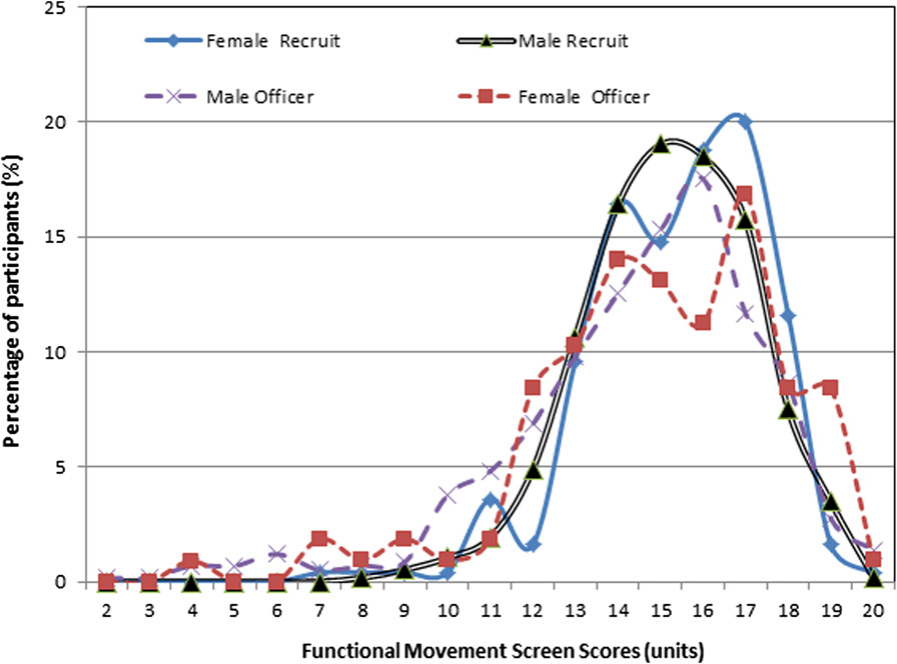
Frequency distributions by group for overall FMS scores

The frequency distributions of FMS total scores plotted by work status (recruit or officer) and gender (male or female) are shown in Fig. 1. Mean ranks of FMS component and total scores and means and standard deviations of FMS total scores are provided in Tables 2 and 3 for the different grouping combinations assessed in the study. Significant differences in distributions of FMS component scores between recruits and serving officers were found for several FMS movement patterns, with recruits also performing significantly better than attested officers on the FMS overall (Table 2). Of note, no statistically-significant differences in distributions of FMS component scores for leg strength (overhead squat, *U* = 273650, *p* = .154, and inline lunge, *U* = 280973, *p* = .738) or leg flexibility (active straight leg raise, *U =* 272307, *p* = .145) were found between the recruits and officers. However, the recruits did significantly outperform the officers in upper limb flexibility (shoulder mobility, *U* = 193921, *p* < .001) and in rotational torso stability (rotary stability, *U* = 259727, *p* < .001), though not in single leg balance, where officers performed significantly better (hurdle step, *U* = 251476, *p* < .001). The recruits also outperformed the officers in torso stability (trunk stability push-up, *p* = .002) and this difference approached, but did not reach statistical significance.

**Table 2:** Mean ranks of component and total FMS scores by qualification and by gender

	Recruits (*n* = 823)	Officers (*n* = 689)	Females (*n* = 357)	Males (*n* = 1155)
Overhead Squat	744.50	770.83	718.52	768.24
Hurdle Step	717.56*	803.01	776.58	750.29
Inline Lunge	753.40	760.20	831.56**	733.27
Shoulder Mobility	865.37*	626.45	949.01**	697.00
Active Straight Leg Raise	742.87	772.78	859.28**	724.73
Trunk Stability Push Up	783.63	724.10	572.76**	813.29
Rotary Stability	785.41*	721.96	773.82	751.15
FMS Total Score	792.54* (Mean ± SD 15.23 ± 2.01)	713.45 (Mean ± SD 14.57 ± 2.96)	810.40 (Mean ± SD 15.24 ± 2.35)	739.84 (Mean ± SD 14.84 ± 2.55)

Significant difference between Recruits and Officers, based on results of Mann–Whitney U tests, * *p* < .001. Significant difference between female and male officers, based on results of Mann–Whitney U tests, **p < .001

**Table 3:** Mean ranks of component and total FMS scores by gender and by qualification

	Female Recruits (*n* = 250)	Female Officers (*n* = 107)	Male Recruits (*n* = 573)	Male Officers (*n* = 582)
Overhead Squat	173.90	190.92	575.49	580.47
Hurdle Step	171.02	197.64	544.60*	610.89
Inline Lunge	176.87	183.98	570.28	585.60
Shoulder Mobility	186.48	161.52	668.90*	488.51
Active Straight Leg Raise	177.34	182.88	554.64	601.00
Trunk Stability Push Up	190.60*	151.91	610.08*	546.42
Rotary Stability	178.88	179.28	608.20*	548.26
FMS Total Score	182.47 (Mean ± SD 15.37 ± 2.04)	170.88 (Mean ± SD 14.93 ± 2.92)	607.25 (Mean ± SD 15.18 ± 1.99)	549.20 (Mean ± SD 14.50 ± 2.97)

Significant difference between Recruits and Officers of equivalent gender, based on results of Mann–Whitney U tests, *p ≤ .001

Comparisons by gender (Table 2) yielded further interesting results, with male personnel significantly poorer performers in the inline lunge (*U* = 179336.5, *p* < .001), but better performers in upper body strength (trunk stability push up, *U* = 140572, *p* < .001). The female participants significantly outperformed the males in flexibility scores (active straight leg raise, *U* = 169475, *p* < .001; shoulder mobility, *U* = 137442.5, *p* < .001).

When comparing the recruits with the officers of their respective genders (Table 3), it was evident that the female recruits exhibited significantly greater strength (trunk stability push up, *U* = 10476, *p* = .001), based on the distributions of mean ranks, when compared to female officers. In other respects, they were similar, though differences in single leg balance (hurdle step, U = 11381, *p* = .006) and shoulder mobility (U = 11505, *p* = .008) approached statistical significance. Similar findings occurred in the male recruits when compared to male officers, who performed better on shoulder mobility (*U* = 114660, *p* < .001) and hurdle step balance (*U* = 147602, *p* < .001). However, the male recruits also outperformed the male officers in trunk stability (trunk stability push up, *U* = 148364, *p* < .001 and rotary stability, *U =* 149436.5, *p* < .001).

ANCOVA, using age as a covariate while assessing the associations between FMS total scores and gender and personnel role, revealed that age was a significant factor (F (1,1507) = 23.519, *p* < .001) in accounting for the observed differences between personnel groups (based on gender and personnel type) in the FMS total scores. When age was controlled in the ANCOVA, gender and personnel role (ie recruit or officer) were not statistically significant influences on the FMS total scores (F(1,1507) = 2.378, *p* = .123 and F(1, 1507) = 0.88, *p* = .766, respectively) in their own rights. A supplementary analysis found that thirty percent of personnel aged 19–20 years and 50% of personnel aged 40–58 years achieved an FMS total score of 14 or less, with the increase steady across the consecutive age groups.

## Discussion

The objective of this study was to profile functional movement capabilities of an Australian state police force. The overall FMS results presented in our study vary from findings in other studies. In our study, FMS total scores across the population (14.93 ± 2.51) were lower than those presenting in other studies of active duty service members (16.2 ± 2.2) [23], Emergency Task Force police officers (15.1 ± 2.1) [24], and an active younger population of people between 18 and 30 years of age (15.7 ± 1.9) [12]. The mean FMS scores in our study were, however, higher than those documented for a Canadian general population (14.14 ± 2.85) [10] and for fire fighters (13.6 ± 1.9) [24] and football players (13.3 ± 1.9) [24].

When comparing recruits to attested officers for FMS total scores, the recruit population performed significantly better (Table 2). While the hurdle step scores were significantly poorer for the recruits when compared to the officers (Table 2), the recruits performed significantly better in shoulder mobility and rotary stability. However, it is evident that the higher mean FMS total scores in recruits when compared to the attested officers is due to the older ages of attested officers, on average. Previous research has found that as the age of the participants increased FMS scores generally decreased [10, 25] and age was similarly found to be a significant factor associated with FMS total scores in this research. These findings regarding a reduction in FMS performance scores with increasing age are understandable given the results of previous research which suggest a decline in both flexibility and strength with increases in age [26]. It should be noted however that this trend may not be conclusive, with other research noting that participants in the 35 to 39 year age group outperformed all other age groups (ages <35, 40–44 and >44 years) in total FMS scores [24]. Although this difference did not reach statistical significance, it does show a variation on the trends noted above and indicates that the relationship between age and FMS total scores may vary with tactical context and population.

In the current study, differences between the genders in FMS total scores did not reach significance. Our findings are consistent with the findings of one study which found no differences between genders in mean FMS total scores [12]. However, our findings differ from those of previous research which have found female participants’ mean FMS total scores to be higher than male mean overall FMS scores [10, 24]. It should be noted, however, that in the current study a trend towards higher scores in female participants was noted and did approach but did not reach significance (*p* = .007). Nevertheless, it is apparent from the ANCOVA results that once age is taken into account, gender is not a contributor to FMS total scores.

When each component of the FMS was examined in isolation (Table 2), female participants were found to have significantly higher scores than male participants in both shoulder mobility and straight leg raise. Conversely, male participants exhibited significantly higher scores for the stability push up (Table 2). These results are consistent with those of previous research [12] and follow general findings of flexibility and strength differences between genders [26].

One notable difference between the findings of our study and findings of previous research [12] was in relation to trunk stability. While our study observed no significant differences between the genders in mean rotary stability (Table 2), males did present with significantly higher mean scores in the earlier research [12]. The reason for this difference is unclear, but may be related to contextual and population differences.

When considered against other studies, the mean female recruit FMS scores found in the current study (Table 3) were similar to the mean FMS total scores for the age stratified range of 20 to 39 years in female Canadian adults [10] (15.37 ± 2.04 and 15.42 ± 2.44 respectively). Conversely, the male recruits in this study presented with a higher mean FMS total score (Table 3) when compared to the Canadian male sample (15.18 ± 1.99 and 14.79 ± 2.76 respectively).

Previous research has identified an increased risk of injuries in both sporting [7, 11] and tactical [19, 20] personnel who achieve FMS total scores of 14 or less. In this study, the mean overall FMS score of all four groups was found to be above 14. However, when the frequency distributions of FMS scores were plotted and examined (Fig. 1), it was found that 33 % (*n =* 82) of female recruits, 36 % (*n =* 204) of male recruits, 41 % (*n =* 44) of female officers and 43 % (*n =* 249) of male officers, scored 14 or less on the FMS. The increased risk levels for male and female officers are reflected in the slightly more pronounced areas under the curve reflected in the left-hand ‘tails’ of the frequency distributions of overall FMS scores for these groups (Fig. 1), and again it should be noted that age differences between the groups appear to be the greatest influence on these between-group differences. Across all groups, 30 % of personnel aged 19–20 years and 50% of personnel aged 40–58 years achieved an FMS total score of 14 or less, with the increase steady across the consecutive age groups.

When each component of the FMS was viewed in isolation, the lowest results were generally achieved in the hurdle step and rotary stability elements – it is notable that these reflect weaknesses corresponding to the leading sites of injury (knee and back) for the police force from which this sample was drawn [3]. In addition, male officers in this study were found to score poorly in the shoulder mobility movement (1.87 ± 0.94). This is of note, given that the shoulder has been found to be a notable site of injury in male officers [3] and poor shoulder range of motion is known to be a potential cause of shoulder injury [27]. As such, the results of this study when considered in the light of the findings of another program of research investigating injuries in this same population [3] suggest a relationship may exist between FMS component scores and injury – a relationship which requires further dedicated research.

While there is limited research on the profiles of injuries in a police force, back injuries are known to be of concern [28, 29]. Long periods of sitting in prolonged and altered postures [28] reaching and rotating across the body to operate a mobile data terminal [29] and the physical restraint of offenders [30] are likely to over stress the back. Considering this, while we have noted a decrease in movement quality of the trunk (as measured by rotary stability) it is important to state that this association may not be causative. Furthermore, the nature of the relationship cannot be ascertained without further study. For example, is it the nature of the tasks leading to reduced rotary stability and potential injury, or is it the reduced rotary stability leading to injury during the task?

A limitation of this study is its isolation to one specific police force within a single state. As such, the results are restricted to the specifics of that police force, like entry fitness standards, roster systems and typically daily tasks. Considering this, the transferability to other law enforcement departments may vary depending on the specifics of that department. Furthermore, it must be acknowledged that while all recruits were required to complete the FMS as part of training, only attested officers who volunteered were assessed. As such, the results for officers may have been skewed towards those who were more physically active. Another limitation of note is the lack of any injury history data which could influence FMS component scores and inform findings regarding specific components of poorer FMS movement performance. Finally, multiple testers were used across the various groups. However, the FMS has been shown to have high inter-rater reliability and all of the staff received formal training on the screening tool (including formal group assessments) at their annual PTI training. As it was not possible to make definitive conclusions regarding associations between FMS scores and injuries in this study, and as such this relationship is hypothesised based on this study and previous studies in regards to injuries, further research in this area is required.

## Conclusion

The findings of this study suggest that attested police officers have a reduced functional movement capability when compared to younger recruits, with a greater percentage of officers scoring 14 or below on the FMS - a score noted for representing an increased risk of injury. Furthermore, the areas of poorest movement performance on the FMS in attested officers aligned with the sites of the body found to be most commonly injured in other research with this population. These findings highlight several opportunities for improvement that can be measured by the FMS. The first is the need for attested officers to maintain their movement skills beyond initial training. The importance of this requirement is highlighted by the absence of any current formal requirement for attested officers to maintain their fitness. Secondly, there may be a benefit of paying attention to specific areas of poorer movement performance for each individual officer and where possible these measures should be monitored for movement performance degradation from initial training as these movement performance areas may be aligned with bodily sites that officers are most likely to suffer and injury. As such, general conditioning programs to maintain functional and specific conditioning programs to improve strength, range of motion and stability in areas of weakness may be of value in mitigating risk of injuries. Female recruits and officers would benefit from conditioning programs that have an increased focus on developing strength while conversely male recruits and officers may benefit from increasing their flexibility. Overall, conditioning programs that lead to improvements in hurdle step and rotary stability (balance and strength), and in shoulder range of motion (stretching and strength through range) may be of benefit to minimise the risk of injury in attested officers.

## Abbreviations

ANCOVA, Analyses of Covariance; FMS, Functional Movement Screen; SPSS, Statistical Package for the Social Sciences.
